# The Gut Microbiome of the Vector *Lutzomyia longipalpis* Is Essential for Survival of *Leishmania infantum*

**DOI:** 10.1128/mBio.01121-16

**Published:** 2017-01-17

**Authors:** Patrick H. Kelly, Sarah M. Bahr, Tiago D. Serafim, Nadim J. Ajami, Joseph F. Petrosino, Claudio Meneses, John R. Kirby, Jesus G. Valenzuela, Shaden Kamhawi, Mary E. Wilson

**Affiliations:** a Departments of Microbiology, Internal Medicine and Epidemiology, University of Iowa, Iowa City, Iowa, USA; b Vector Molecular Biology Section, Laboratory of Malaria and Vector Research, National Institute of Allergy and Infectious Diseases, National Institutes of Health, Rockville, Maryland, USA; c Department of Molecular Virology and Microbiology, Alkek Center for Metagenomics and Microbiome Research, Baylor College of Medicine, Houston, Texas, USA; d Iowa City Veterans’ Affairs Medical Center, Iowa City, Iowa, USA; Washington University School of Medicine; National Institute of Allergy and Infectious Diseases

## Abstract

The vector-borne disease leishmaniasis, caused by *Leishmania* species protozoa, is transmitted to humans by phlebotomine sand flies. Development of *Leishmania* to infective metacyclic promastigotes in the insect gut, a process termed metacyclogenesis, is an essential prerequisite for transmission. Based on the hypothesis that vector gut microbiota influence the development of virulent parasites, we sequenced midgut microbiomes in the sand fly *Lutzomyia longipalpis* with or without *Leishmania infantum* infection. Sucrose-fed sand flies contained a highly diverse, stable midgut microbiome. Blood feeding caused a decrease in microbial richness that eventually recovered. However, bacterial richness progressively decreased in *L. infantum-*infected sand flies. *Acetobacteraceae* spp. became dominant and numbers of *Pseudomonadaceae* spp. diminished coordinately as the parasite underwent metacyclogenesis and parasite numbers increased. Importantly, antibiotic-mediated perturbation of the midgut microbiome rendered sand flies unable to support parasite growth and metacyclogenesis. Together, these data suggest that the sand fly midgut microbiome is a critical factor for *Leishmania* growth and differentiation to its infective state prior to disease transmission.

## IMPORTANCE

*Leishmania infantum*, a parasitic protozoan causing fatal visceral leishmaniasis, is transmitted to humans through the bite of the sand fly *Lutzomyia longipalpis*. Development of the parasite to its virulent metacyclic state occurs in the sand fly gut. In this study, the microbiota within the *Lu. longipalpis* midgut was delineated by 16S ribosomal DNA (rDNA) sequencing, revealing a highly diverse community composition that lost diversity as parasites developed to their metacyclic state and increased in abundance in infected flies. Perturbing sand fly gut microbiota with an antibiotic cocktail, which alone had no effect on either the parasite or the fly, arrested both the development of virulent parasites and parasite expansion. These findings indicate the importance of bacterial commensals within the insect vector for the development of virulent pathogens, and raise the possibility that impairing the microbial composition within the vector might represent a novel approach to control of vector-borne diseases.

## INTRODUCTION

The *Leishmania* spp. are vector-borne protozoan parasites that cause a group of highly prevalent and neglected tropical diseases called leishmaniasis (1). The parasites are transmitted to mammalian hosts through a bite of an infected female phlebotomine sand fly. Leishmaniasis is prevalent among poor populations in the 98 countries and territories where it is endemic (1). The visceral form of leishmaniasis is spreading among dense and growing populations in periurban areas of Brazil (2). Although wild reservoir hosts can maintain the infection, the domestic dog is thought to be a major reservoir in the northeast of Brazil, facilitating transmission to humans in peridomestic settings (3). Development of a successful preventive vaccine for human leishmaniasis has been elusive; therefore, additional measures to curtail vector-mediated transmission in areas of endemicity could be of tremendous benefit (4–7).

The extracellular promastigote form of *Leishmania* parasites resides and develops to its infectious metacyclic form solely in the gut lumen of the sand fly (8). Only specific sand fly species-*Leishmania* species combinations are successful in yielding metacyclic parasites capable of transmitting infection to mammalian hosts (9). In several cases (e.g., *L. donovani—Phlebotomus argentipes*, *L. major—P. papatasi*, *L. tropica—P. sergenti*), this pairing has been shown to correlate with the ability of promastigote surface lipophosphogylcan to interact with the sand fly gut (10–12). Within the gut of the sand fly vector, *Leishmania* parasites taken up with a blood meal must escape the peritrophic matrix and attach to midgut epithelium (8, 13, 14). Parasites replicate and develop through several gut-adherent forms as they progress toward the anterior end of the gut. Eventually, parasites develop to a nondividing, nonadherent, and infectious metacyclic life stage ready for inoculation into the mammal (8, 15). The current report is based on the observations that insect guts are usually inhabited by a community of commensal bacteria (16) and that the gut microbiomes of mosquitoes and tsetse flies have been found to influence the competence of these insects as vectors of parasitic disease (13, 17, 18). We hypothesized that the midgut bacterial microbiome might in part determine whether sand flies can support the development of infectious *Leishmania* species parasites, capable of being transmitted to a mammalian host.

The goal of the current study was to characterize the specific bacterial microbiome of sand fly midguts during *Leishmania* species infection, and their potential influence on the biology of the parasite within its insect vector host. Prior studies of sand fly-associated microbiota have utilized different methods and thus address different issues. Some studies have taken a culture-based approach that yields useful information but limits the findings to only bacteria that can be recovered from *in vitro* cultures (19, 20). One study performed 16S ribosomal DNA (rDNA) sequencing on genomic DNA extracted from whole sand flies, including the exoskeleton and other body parts, and did not distinguish midgut from surface microbiota (21). A more comprehensive 16S rDNA sequencing approach was taken by a group studying uninfected flies (22). These findings are all valid, but they do not provide a view of the complexity of the midgut microbiome surrounding the developing parasite. Therefore, we adopted a comprehensive approach to define the microbiota in the sand fly gut using 16Sv4 rDNA gene sequencing to examine the structure, diversity, and temporal dynamics of the colony-reared sand fly gut microbiome over time in response to a blood meal with or without *L. infantum* infection. The data showed that the composition of the sand fly gut microbiome is dynamic and responds to the feeding habits of the sand fly and to the parasite itself. Furthermore, antibiotic treatment revealed an important role for the sand fly gut microbiome in restricting *L. infantum* replication and the subsequent development of infectious metacyclic parasites within the sand fly vector. Thus the midgut microbiome in part determines the infection capacity of the parasite.

## RESULTS

### Composition and diversity of the gut microbiome in sand flies.

The variation in the diversity of the sand fly gut microbiome was determined under conditions designed to model those encountered by field flies. Sand flies were fed sucrose or a blood meal (blood-fed sand flies) or a blood meal combined with *L. infantum* amastigotes (*L. infantum-*infected sand flies).

**FIG 1:**
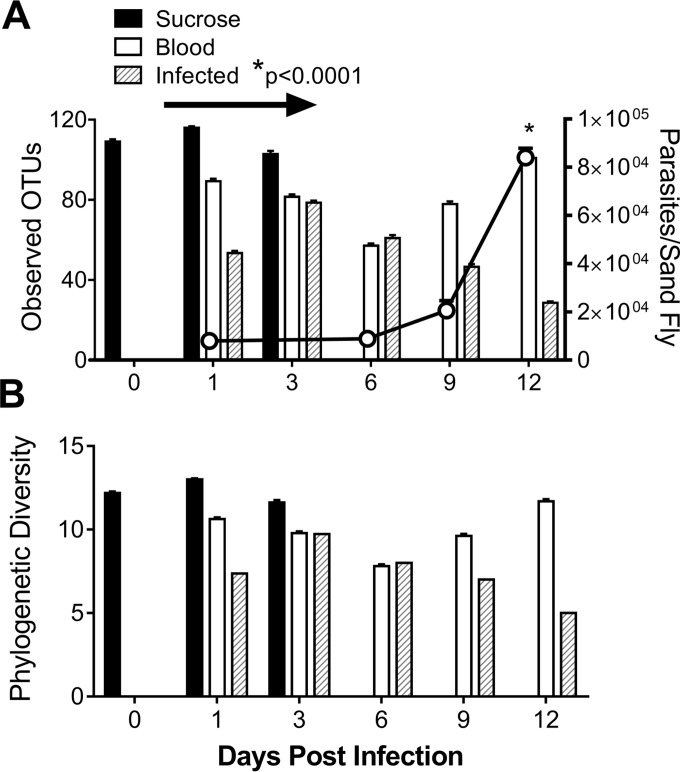
*L. infantum* parasite burden is inversely correlated with bacterial richness in infected sand fly midguts, measured in numbers of OTUs at the species (A) or phylum (B) level. (A) The total number of OTUs obtained by bacterial 16S rDNA sequencing of DNA derived from the gut of sand flies fed on different meals (bars, left *y* axis) was determined in triplicate pools of 20 sand fly midguts under each condition, in three separate biological replicate experiments. The parasite burden represents the mean of results of 3 separate experiments in which parasites in 10 infected flies from each condition were counted microscopically. Panel A (line graph, right *y* axis) shows the means ± standard errors (SE) of the results from 3 replicate experiments. *, 2-way ANOVA with Tukey posttest was performed between treatments over time. The arrow indicates the duration of detectable blood in sand flies fed on blood. (B) UniFrac diversity metric over time in each treatment group. Sucrose-fed flies were obtained for the day 0 to day 3 time points. Data for days 0 to 9 are from three independent experiments; data for day 12 are from two independent experiments. Each experiment included triplicate pools of 20 dissected sand fly midguts.

Results of 16S rDNA sequencing were analyzed for each experimental condition on different days of the experiment. Equal numbers of fly midguts were included in all replicate samples of each treatment group at each time point to ensure that the treatment groups were comparable. The total number of observed operational taxonomic units (OTUs) present in the sequenced microbiome is indicative of bacterial richness (Fig. 1A) based on the number of different OTUs identified within a treatment group. In contrast, bacterial phylogenetic diversity takes into consideration the phylogenetic relationship between the treatments at each time point (Fig. 1B). Sand flies fed only sucrose harbored the highest number of OTUs compared to blood-fed or *L. infantum-*infected sand flies (Fig. 1A). The blood meal was observed to have been digested by day 6, after which the levels of bacterial diversity in blood-fed versus *L. infantum-*infected sand flies diverged. After the disappearance of the blood meal (arrow in Fig. 1A), the bacterial richness (number of distinct OTUs) in blood-fed flies recovered to the level seen with the sucrose-fed controls (Fig. 1A, filled bar), indicating that blood can impact the community diversity in a reversible fashion. In contrast, bacterial richness continued to diminish in *L. infantum*-infected sand flies as the parasite load increased (*P* < 0.0001) (Fig. 1A), indicating that *L. infantum* can impact the composition of the gut microbiota.

Beta diversity, measured by UniFrac analyses (23), represents the explicit comparison of distances between bacterial communities based on their phylogenetic composition. The UniFrac data representing variants between samples are either weighted (quantitative data), considering the abundance of observed OTUs, or unweighted (qualitative data), considering only the presence or absence of OTUs. In the current study, the results of distance comparisons of all treatment groups and all time points showed statistical significance (see Fig. S1A in the supplemental material) (distance results are displayed as principal-component data in Fig. S1). The diversity between samples from *L. infantum*-infected sand flies at different times of the infection accounted for this significance (*P* = 0.023) (Fig. S1B). There were no significant differences in the diversity of bacterial communities in sucrose-fed or blood-fed flies at different times (*P* > 0.05) (Fig. S1C and D).

10.1128/mBio.01121-16.1 Figure S1  The diversity of microbial communities between different time points. Diversity between conditions was determined by weighted and unweighted UniFrac analyses to indicate differences between bacterial genus OTUs in communities of sucrose-fed, blood-fed, or *L. infantum-*infected blood-fed sand fly midguts over time. Results of principal-component analyses are shown for all time points in midguts of sand flies under all conditions (A), sand flies fed on sucrose alone (B), sand flies receiving a blood meal (C), or sand flies fed on blood plus *Leishmania infantum* (D). Download Figure S1, TIF file, 2 MB. Copyright © 2017 Kelly et al. 2017 Kelly et al. This content is distributed under the terms of the Creative Commons Attribution 4.0 International license.

**FIG 2:**
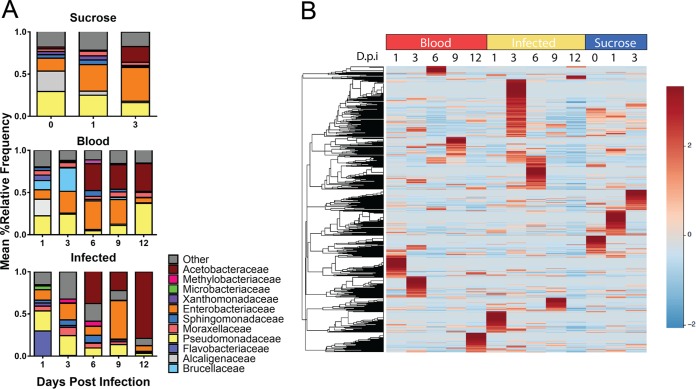
Temporal variation in the relative abundances of midgut OTUs. (A) Bar graphs represent the relative abundances of family-level OTUs in sand flies under each condition. OTUs representing >5% of the overall relative abundance under at least one condition are shown; OTUs that remained at less than 5% of the overall abundance under all conditions are pooled as “other.” (B) A heat map of species-level OTUs over time in all treatment groups, clustered by Euclidean distances, is shown. Data for days 0 to 9 are representative of three independent experiments; data for day 12 are from two independent experiments. Sequence data were obtained from the same 3 experiments described for Fig. 1.

The relative abundance of OTUs in the sand fly gut microbiome was monitored throughout 12 days of parasite development (Fig. 2). OTUs present in ≥5% of samples were widely divergent between all treatment groups and over time in infected flies. By day 12, *Pseudomonadaceae* comprised only 3% of the bacterial microbiome in *L. infantum-*infected sand flies compared to 36% in blood-fed sand flies (Fig. 2A). Conversely, 78% of the gut microbiome in infected sand flies at day 12 belonged to the family *Acetobacteraceae*, compared to 33% in blood-fed controls. The relative abundances of all OTUs are listed in Table S1 in the supplemental material. Several species previously identified in field-caught sand flies, such as *Erwinia* spp. and *Asaia* spp., were also found in our laboratory-reared sand flies (Fig. S2) (20, 21, 24). The variation in the relative abundance of OTUs under each condition over time is illustrated in the heat map in Fig. 2B.

10.1128/mBio.01121-16.2 Figure S2  Lack of fungal outgrowth in antibiotic-treated sand flies. DNA was extracted from four replicate pools of 20 sand fly midguts each and was analyzed for the presence of fungal DNA by qPCR of 18S primers. Sand fly midguts were harvested at day 9 after *L. infantum* infection. Files were either fed sucrose alone (control) or fed on sucrose with antibiotics at day 6. Data were compared to a standard curve (10^2^ to 10^8^ counts) for a DNA fragment based on fungal 18S sequence. Download Figure S2, TIF file, 0.2 MB. Copyright © 2017 Kelly et al. 2017 Kelly et al. This content is distributed under the terms of the Creative Commons Attribution 4.0 International license.

10.1128/mBio.01121-16.3 Figure S3  Relative abundances of specific bacterial genera differ over time between treatment groups. Data represent relative abundances of bacterial taxonomic units in sucrose-fed, blood-fed, or *L. infantum-*infected blood-fed sand fly midguts over time. OTUs were selected in QIIME. Data are representative of results from three independent experiments. Download Figure S3, TIF file, 0.7 MB. Copyright © 2017 Kelly et al. 2017 Kelly et al. This content is distributed under the terms of the Creative Commons Attribution 4.0 International license.

10.1128/mBio.01121-16.4 Table S1  Taxononomic categories are shown for each species represented in the microbiomes under at least one condition tested in this study. Data show the means ± numbers of OTUs corresponding to each species in the three repeat experiments, each with *n* = 3 replicate samples for each time point. Total numbers at the bottom of the table indicate the total mean OTUs under each time point/condition. Download Table S1, XLSX file, 0.1 MB. Copyright © 2017 Kelly et al. 2017 Kelly et al. This content is distributed under the terms of the Creative Commons Attribution 4.0 International license.

**FIG 3:**
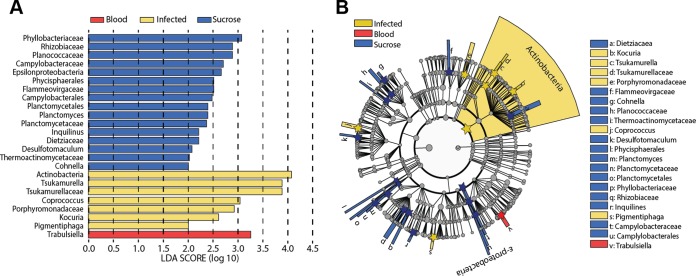
The “uniqueness” and divergence of the microbiome under each sand fly condition determined by linear discriminant analysis. (A) Multiple regression results for Fisher’s linear discriminant analysis (LDA) of microbiomes from each of the sand fly diets, utilizing discriminatory OTUs to define phylogenetic levels. LDA is used to determine the effect size and identify components of the bacterial kingdom microbiomes determining the “uniqueness” of each sand fly group. Colors indicate the fly condition of the phylogenetic component contributing to group uniqueness at an LDA score of >2.0. (B) Cladogram visualization of the LDA results from the experiment represented in panel A. Levels of the cladogram represent, from the inner to outer rings, phylum, class, order, family, and genus. Color codes indicate the condition, and letters indicate the taxa that contribute to the uniqueness of the corresponding sand fly group at an LDA of >2.0. Sequence data were obtained from the same 3 experiments described for Fig. 1.

A linear discriminant analysis (LDA) of effect size was performed to determine the OTUs that discriminate between each of the sand fly conditions. The data revealed that members of the *Actinobacteria* phylum account significantly for the divergence of the microbiome of *L. infantum-*infected sand flies from the microbiome of either blood-fed or sucrose-fed sand flies (Fig. 3A). The *Trabulsiella* family distinguished blood-fed sand flies from the other groups. Sucrose-fed sand flies had many taxa that were distinct at this level, the most significant of which was the family *Phyllobacteriaceae*. The OTUs represented in each experimental group are illustrated at a variety of phylogenetic levels from phylum to genus as a cladogram in Fig. 3B.

### Predicted metagenomic analyses revealed various degrees of nutrient acquisition in the sand fly midgut microbiome.

**FIG 4:**
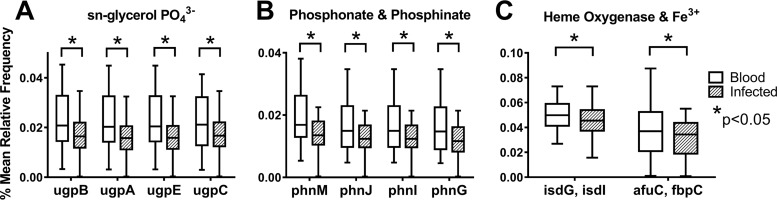
KEGG orthologs of the iron and phosphate ATP-binding cassette (ABC). Transport pathways differ between blood-fed and *L. infantum-*infected sand flies. (A to C) Box and whisker plots illustrate the predicted metagenomic content of ABC transport orthologs defined in KEGG. The functional metagenomes were calculated from OTUs with PICRUSt, and the ortholog content in the microbiomes of midguts from uninfected versus *L. infantum-*infected blood-fed sand fly midguts was determined by PICRUSt. Significant orthologs were selected by one-way ANOVA and the Tukey-Kramer posttest. All statistically significant pathways were determined using one-way ANOVA and the Tukey-Kramer posttest (*, *P* < 0.05). Data for uninfected and *L. infantum*-infected blood-fed sand flies include all samples from 1 to 12 dpi. Sequence data were obtained from the same 3 experiments described for Fig. 1.

We utilized PICRUSt and STAMP to identify putative predicted functional pathways present in bacterial microbiomes in midguts of sand flies under the different conditions. Pathways and relevant orthologs that were significantly enriched based on their presence within the genomes were identified using the Kyoto Encyclopedia of Genes and Genomes (KEGG) database (25). Among the most significantly enriched pathways were two with high levels of representation of significantly expressed KEGG orthologs. Levels of pathways that allow microbes to acquire PO_4_^3−^ and Fe^3+^ via ATP-binding cassette (ABC) transport systems were predicted to be significantly lower (*P* < 0.005) in the microbiota of *L. infantum-*infected sand flies than in the microbiota of blood-fed sand flies (Fig. 4).

### A decrease in midgut microbiota impaired *Leishmania* survival in the sand fly.

**FIG 5:**
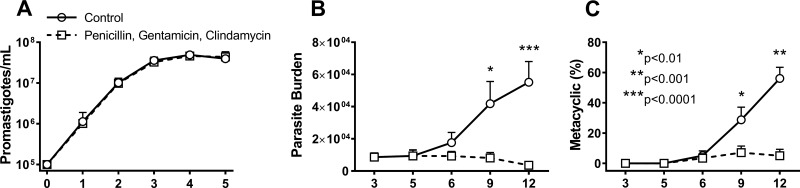
Antibiotic treatment of infected sand flies halts parasite survival and replication and development of metacyclic forms. (A) *In vitro Leishmania infantum* growth curve in the absence or presence of antibiotics. Data are representative of results from three independent replicates. (B and C) *In vivo Leishmania infantum* growth curve (B) and metacyclic parasite counts (C) in the absence and presence of antibiotics during sand fly infection over time. Data are representative of results of four independent biological replicates. Antibiotics included a cocktail of penicillin, gentamicin, and clindamycin. The arrow indicates the day of antibiotic treatment. Statistical analyses included Student’s *t* test (*, *P* < 0.01; **, *P* < 0.001; ***, *P* < 0.0001) and two-way ANOVA (***, *P* < 0.0001)

To determine whether the interaction between the microbiome and *L. infantum* is beneficial or detrimental for the parasite, we fed a group of day 5 infected sand flies daily on sucrose containing an antibiotic cocktail of penicillin, gentamicin, and clindamycin to deplete the native microbiome. Control *L. infantum-*infected sand flies were maintained on sucrose without antibiotics. A preliminary study determined that the antibiotics did not affect the growth or the morphological development of *L. infantum* to metacyclics in culture (Fig. 5A) but did inhibit growth of a majority of the recoverable sand fly gut bacteria, which showed a decrease from 2,772 to 10 CFU per gut cultured on LB agar plates. Midguts were dissected 1, 4, or 7 days after taking a meal that included the antibiotic cocktail (days 6, 9, and 12 of the experiment). 18S rDNA sequences specific to fungi were amplified from samples of untreated or antibiotic-treated day 9 infected sand flies. The abundance of the product was compared to an 18S standard curve. Both no-template controls (NTC) and fungal 18S sequences from four biological replicates, each tested in duplicate, revealed threshold cycle (*C*_*T*_) values near the lower end of the standard curve. Differences between the abundance of 18S product in the untreated versus the antibiotic-treated sand fly samples were not statistically significant (paired *t* test; Fig. S2).

Quantification of parasites in midguts from sand flies revealed that parasite replication was impaired after only 24 h following antibiotic treatment compared to the level seen with control untreated infected sand flies (Fig. 5B). Daily successive treatment with antibiotics inhibited both parasite growth and development to infectious metacyclic forms. Proportions of metacyclics were significantly lower on days 9 (*P* value < 0.005) and 12 (*P* value < 0.001) in antibiotic-fed flies than in control infected sand flies (Fig. 5C). Thus, development of mature transmissible infections was efficient in sand flies harboring a normal bacterial microbiome but was severely impaired in antibiotic-treated sand flies.

## DISCUSSION

Bacteria present in insect vectors of infectious diseases can affect their capacity to transmit infection (17). The *Leishmania* species parasites reside, replicate, and develop from an avirulent to a virulent state entirely within the midgut of the sand fly vector. It therefore seems highly likely that microbiota present in the midgut would interact with and consequently influence the metabolism and virulence of developing *Leishmania* species protozoa. To address this hypothesis, we undertook comprehensive 16S rDNA gene high-throughput sequencing of DNA recovered from isolated midguts of infected or uninfected *Lutzomyia longipalpis*, the vector transmitting *Leishmania infantum*. Analysis of the bacterial phylogenies present in 2,091 isolated midguts of colony-reared *Lu. longipalpis* revealed a surprisingly diverse array of bacterial families, which differed on the basis of the source of the insect’s meal and infection status. Controlling for these changes, the presence of *L. infantum* infection caused a progressive and dramatic loss of bacterial diversity as the infection progressed (20, 21, 24). Furthermore, antibiotic-mediated eradication of midgut bacteria had a severe negative impact on *Leishmania* replication and, consequently, on development to the infectious metacyclic form. Due to the lack of metacyclics in antibiotic-treated flies, one would predict that these insects would not be able to transmit a productive infection to a host.

The composition of the midgut bacterial microbiome varied over time in each experimental group. Bacterial diversity in the blood-fed group reverted toward that of the sucrose-fed group, whereas the diversity of infected midguts decreased over time, and on day 12 it was largely composed of members of the family *Acetobacteraceae*. According to weighted and unweighted UniFrac analyses, there were no significant differences between replicate samples, but there were several bacterial phylogenetic groups in each of the groups that uniquely distinguished the treatment groups at each time point. Results of multiple-regression LDA determined that bacteria within the phylum *Actinobacteria* significantly distinguished the microbiome of infected sand flies from that of uninfected controls fed on either blood or sucrose. Similarly, members of the family *Phyllobacteraceae* and the genus *Trabulsiella* distinguished the midgut microbiomes of sucrose-fed and blood-fed sand flies, respectively. These data underscore the fact that the sand fly gut microbiome is dynamic and responsive not only to the type of meal but also to the absence or presence of *Leishmania* species infection.

Previous studies of the microbiomes of sand flies transmitting *Leishmania* species infections have utilized a variety of methods, including denaturing gradient gel electrophoresis (DGGE), bacterial culture, or 16S rDNA/total high-throughput sequencing (19, 21). DGGE analysis of DNA extracted from *Lu. longipalpis* or *Lu. cruzi* from rural or sylvatic Brazil or Colombia revealed the *Proteobacteria* *Erwinia* and *Ralstonia* spp. among other bacteria (24), both of which were also present in our samples. Monteiro et al. sequenced the midgut metagenomes of *Lu. intermedia*, a vector transmitting *L. braziliensis*, in pooled midguts from unfed, blood-engorged, or gravid sand flies. Although the infection status of the flies was not taken into consideration, that study is the one most directly comparable to our current report. *Enterobacteriacae* was the most abundant family in the uninfected and gravid groups but represented only 4.2% of the bacterial families of blood-fed groups. In contrast, we observed prominent *Enterobacteriacae* under both sucrose-fed and blood-fed conditions. The *Enterobacteriaceae* were overtaken by *Acetobacteraceae* on day 12 of infection, a condition not included in the Monteiro study. Monteiro et al. observed *Rickettsiaceae* as the most abundant family in the *Lu. intermedia* blood-fed fly pool, and among the *Rickettsiaceae* family members the *Wolbachia* genus represented 46.7% of the sequences. This is in stark contrast with our study of *Lu. longipalpis*, in which *Wolbachia* spp. were identified only in sucrose-fed flies and not in blood-fed or infected flies. Other bacterial genera in the *Lu. intermedia* study included members of the phyla *Proteobacteria* and *Brucellaceae*, all of which were represented in our current study of *Lu. longipalpis*. Another commonality was *Pseudomonas* (phylum *Proteobacteria*), which was prominent in *Lu. intermedia* and present in all of our samples (22). *Pseudomonas* was actually one of the most variable genera between conditions in our study, with levels diminishing as the levels of both the family *Acetobacteraceae* and the *L. infantum* infection increased. Whether the lowered pH due to acetic acid release from *Acetobacteraceae* influenced the parasite development is an intriguing issue that would bear further pursuit (26).

A few studies associated the capacity of sand flies to serve as vector hosts for *Leishmania* spp. with functional consequences of the presence of particular bacteria residing in the sand fly gut (27, 28). Using conventional bacterial cultures, Sant’Anna et al. showed that bacterial infection of *Lu. longipalpis* with *Serratia marcescens* diminished subsequent colonization with *Leishmania mexicana*, potentially via reactive oxygen species (ROS) generation. Conversely, *L. mexicana* infection protected flies from *Serratia marcescens*-induced mortality (27). Using sequence analysis rather than culture, we also observed *Serratia marcescens* in colony-raised *Lu. longipalpis*. In the same theme, our current study using 16S rDNA sequencing to identify the entire bacterial microbiome showed that the total number of OTUs (including *S. marcescens* OTUs) is inversely correlated with total *L. infantum* parasite counts. The inverse relationship between bacterial richness and number of parasites was most evident in examining parasite development over 12 days post-sand fly infection, when the majority of parasites are in the highly infectious metacyclic life stage (15). These data suggest that the hypothesis raised in the publication by Sant’Anna et al., that bacteria such as *S. marcescens* confer resistance to *Leishmania* species infection, warrants further investigation (27). It will be particularly important to address whether these correlations are associated with enhanced microbicidal activity (e.g., ROS) within the gut.

Antibiotic suppression of the native gut microbiome resulted in a dramatic arrest of both parasite replication and metacyclogenesis. Importantly, antibiotics did not perturb the health of the sand fly or the fitness of the parasite itself. There were not concomitant changes in the copy numbers of fungal 18S rDNA genes, suggesting that the effect on the differentiation and expansion of *Leishmania* within the vector was most likely due to decreased bacterial abundance. The data do not reveal whether specific bacterial genera or the total bacterial load is necessary for *L. infantum* development. This role for sand fly vector gut bacteria is the opposite of that predicted by studies of *Anopheles gambiae* mosquitoes harboring *Plasmodium falciparum*, in which antibiotic treatment caused a significant increase in the total number of oocysts (29). Also opposite to our observation, infection of colony-reared mosquitoes with the bacterial symbiont *Wolbachia* can lead to a decrease in numbers of *Plasmodium* parasites in the mosquito vector (30).

Analysis of the predicted metagenome comparing functional pathways differentially expressed by bacteria within the different treatment groups enabled us to generate hypotheses about the mechanisms through which bacterial depletion might affect *L. infantum* development (31). Particularly notable were ABC (ATP-binding cassette) proteins, transporter pathways that are important for nutrient acquisition. The local concentrations of specific nutrients have documented effects on *Leishmania* species differentiation to a virulent state. For instance, iron acquisition is important for *Leishmania* species growth and differentiation *in vitro*, involving the coupled activities of a membrane-associated ferric reductase (*LFR1*) and a ferrous iron transporter (*LIT1*) (32–36). Another example is adenosine, which activates the ABC transporter systems that in turn suppress *Leishmania* species metacyclogenesis (37). We hypothesize that local bacterial genera in the vector provide the signals that trigger parasite differentiation either directly, by altering the concentration of key nutrients that trigger parasite differentiation, or indirectly, through effects on sand fly epithelial cell biology. Sand fly epithelial cells are immunoregulatory, responding to stimuli with Imd or Toll immune response pathways (38–39). Similar to other insect vectors of disease, the sand fly epithelial environment could support or disallow parasite survival (40–43).

These reciprocal effects could highlight a mutual competition between parasites and commensal bacteria for nutrient acquisition. Exemplified by the fact that adenosine deprivation stimulates metacyclogenesis (37), the local nutrient concentration may provide the necessary signals for *Leishmania* differentiation. Our data clearly indicate that specific microbial content is vital for *L. infantum* parasites to survive and successfully differentiate to an infective stage in the sand fly midgut, although the bacterium-associated biochemical mechanisms that trigger *Leishmania* replication and metacyclogenesis remain elusive. It must be emphasized that our study was performed on colony-reared sand flies fed on sterile blood or sucrose or an infected blood meal. It is very likely that midguts from sand flies feeding on wild plants and mammals would differ phylogenetically from those in our study, although similarities between our findings and those of Monteiro et al. suggest that at least some genera may be common (22). In future studies, it will be essential to determine by sequence analysis the similarity between the midgut microbiomes of laboratory-reared sand flies and wild-caught sand flies from neighborhoods where *L. infantum* infection is endemic and whether some bacteria can substitute for others in supporting the growth and differentiation of *Leishmania* spp. The mechanism(s) by which the bacterial microbiome promotes or suppresses *Leishmania* development may be key to understanding the vital role of sand flies in the development and transmission of *Leishmania* spp. to a mammalian host.

## MATERIALS AND METHODS

### Parasites and sand fly infection.

A strain of *Leishmania infantum* originally isolated from a dog spleen in Natal, Brazil (*MCAN/BR/09/52*), was maintained by serial passage in hamsters. *Lutzomyia longipalpis* sand flies, Jacobina strain, were reared at the Laboratory of Malaria and Vector Research, NIAID, NIH. Sand flies were maintained in paper containers at 25°C and 75% relative humidity on a 12-h light/dark cycle. Amastigotes were isolated from hamster spleens, washed in phosphate-buffered saline (PBS), and suspended at 5 × 10^6^/ml in defibrinated rabbit blood.

Female *Lu. longipalpis* sand flies were initially maintained on 30% sucrose provided in cotton swabs. On day 0 of the experiment, sand flies (3 to 5 days old) were divided in 3 groups which were fed one of the three meals: (i) 30% sucrose; (ii) sterile, uninfected rabbit blood; or (iii) *L. infantum* amastigote-inoculated rabbit blood. Sand flies were blood fed through a chick membrane bound to a glass feeder in the dark as previously described (4). After 4 h, sand flies in all groups were shifted back to 30% sucrose cotton swab feedings that were performed daily for the duration of the experiment.

### Sand fly midgut dissection.

Sand fly midguts were dissected on the day of infection (day 0) and at 1, 3, 6, 9, and 12 days postinfection (dpi). Sucrose-fed sand flies did not survive well at later time points and were analyzed at up to 3 days postemergence, when field-caught females take a blood meal. Microscopy and dissection tools were sterilized before midgut dissection. Sucrose-fed, blood-fed, and amastigote-containing-blood-fed groups of sand flies were rinsed in PBS, and individual sand flies were dissected in a sterile pool of PBS on a glass slide. Intact dissected midguts were washed in a pool of sterile PBS five times and then added to 20 µl PBS. For sequence analyses, midguts from 20 flies were pooled and triplicate pools for each condition at each time point were prepared for a total of 60 midguts per condition per time point in each experiment. Three independent experiments were performed. Samples were stored at −80°C until DNA extraction.

*Leishmania infantum* parasite counts were done for a minimum of 10 individual sand flies for each “infected” condition in each experiment. Data in Fig. 1 show the means ± standard errors of results from three replicate experiments. Infection was verified in sand flies used for DNA sequence analysis by visualizing parasites on the dissecting microscope.

### Samples collected for sequence analysis.

Samples collected from each of three independent experiments are enumerated in Table 1. With the exception of day 12, three pools of 20 midguts were collected at each time point of each experiment and used as triplicate samples for sequencing. Due to availability and survival, day 12 sand flies were included in only 2 of the 3 experiments, and only duplicate blood meal or 1 to 2 infected blood meal samples could be collected. The three biological replicates per experiment were independently sequenced, resulting in a total of 9 replicate sequenced samples per condition on all but day 12. Overall, a total of 109 samples were subjected to sequence analysis, including all the replicates described above plus 3 sugar-fed controls.

**TABLE 1:** Experimental design showing the conditions and time points at which sand fly midgut pools were collected for sequencing^a^

Condition	No. of midguts × no. of samples at indicated day postinfection					
	0	1	3	6	9	12^b^
Sucrose fed	20 × 3^c^	20 × 3	20 × 3			
Blood fed		20 × 3	20 × 3	20 × 3	20 × 3	20 × 2
*L. infantum* infected		20 × 3	20 × 3	20 × 3	20 × 3	20 × 2

a Each cell of the table indicates the number of midguts collected under that condition at that time × the number of replicate samples taken. The full table indicates the design for each one of the three independent experiments. All replicate samples were sequenced individually, resulting in 9 sequences per condition at all time points but day 12.

b Limited by survival/availability, two of three independent experiments included day 12 samples. For the blood-fed condition, two replicates per experiment were collected; for the infected-blood-fed condition, one and two replicates, respectively, were collected in the two experiments.

c Twenty midguts were pooled into three independent biological replicates per time point for each condition.

### DNA extraction.

Genomic DNA was extracted using an Omega Bio-Tek tissue DNA extraction kit as described by the manufacturer (Omega, Norcross, GA). Total genomic DNA was quantified on a NanoDrop 2000 spectrophotometer.

### 16S or 18S rDNA amplification and Illumina MiSeq sequencing.

16S rDNA gene sequencing was performed at the Alkek Center for Metagenomics and Microbiome Research, Baylor College of Medicine, Houston, TX, using methods developed for the NIH-Human Microbiome Project (44). The 16S rDNAv4 region was amplified and sequenced on the Illumina MiSeq platform (San Diego, CA, USA) using the paired-end protocol for 2 × 250-bp paired-end overlapping reads (45). A total of 540,000 total reads were generated post-quality filtering, with an average of 4,800 reads per sample.

Quantitative PCR (qPCR) sample analysis was performed in a QuantStudio 7 Flex Real-Time PCR system using a MicroAmp Fast Optical 96-well plate and MicroAmp optical adhesive film (Applied Biosystems). Each reaction mixture contained fungal 18S primers (18S-F [TAAACTATGCCGACTAGGGATCG] and 18S-R [ACTTTGATTTCTCGTAAGGTGCCGA]; Integrated DNA Technologies, Inc., Coralville, IA); PerfeCTa SYBR green FastMix, low ROX (Quanta Biosciences, Beverly, MA); and 1 ng of DNA. Amplification was comprised of a 10-min activation step at 95°C, followed by 40 cycles of 95°C for 10 s and 60°C for 30 s and a fluorescence measurement. Melting curve analysis was done by monitoring fluorescence throughout incremental increases of temperature from 60°C to 95°C. *C*_*T*_ values obtained from experimental samples were compared to a standard curve generated from serially diluted 18S gBlocks gene fragments (Integrated DNA Technologies, Inc., Coralville, IA) to assess fungal outgrowth in the presence or absence of antibiotics. All samples were run in triplicate, including the standard curve and a set of no-template controls (NTC).

### Bioinformatics analyses with QIIME.

The bioinformatics analyses were carried out at the University of Iowa. 16S data were analyzed using QIIME version 1.9 software (46). Barcodes were matched to FASTQ files, after which barcode and primer sequences were removed. Sequences with a minimum pairwise identity of 97% were combined into operational taxonomic units (OTU) using sumaclust v 1.1.00 and sortmerna 2.0 (47). Representative sequences for each OTU were aligned using PyNAST (48). lanemaskPH was used to screen out the hypervariable regions, and OTUs were classified into known taxonomy units using the Greengenes (49) database. Observed species, Chao1, and phylogenetic diversity metrics were assessed with alpha_diversity.py and compare_alpha_diversity.py in QIIME. Beta diversity was assessed with both weighted and unweighted UniFrac metrics using beta_diversity_through_plots.py. A genus-level relative abundance table was utilized to generate heat maps clustered by Euclidean distance in R, using the Bioconductor packages RColorBrewer, Heatmaps2, and vegan.

### Putative metagenome identification.

To infer putative metagenomes from 16S rDNA-generated gene profiles, reads were binned into OTUs at 97% sequence identity using a closed reference strategy utilizing the Greengenes database for identification of OTUs. Phylogenetic Investigation of Communities by Reconstruction of Unobserved States (31) (PICRUSt) software was used to transform the counts of these OTUs into metagenome prediction counts of functional genes for each sample. The STAMP statistical tool was utilized to determine significant putative KEGG orthologs and pathway analyses (50). One-way analysis of variance (ANOVA), two-way ANOVA, and Tukey-Kramer posttest statistical tests were used to compare treatment groups in STAMP-predicted metagenomic analyses.

### Antibiotic treatment.

For a set of experiments, an antibiotic cocktail of penicillin (100 units/ml), gentamicin (50 μg/ml), and clindamycin (4 μg/ml) was included in sucrose meals offered to infected sand flies in 30% sucrose cotton swabs beginning at day 5. Swabs were changed daily throughout the duration of the 12-day experiment. Bacterial growth from midguts recovered from sand flies fed for 24 h on sucrose without or with the antibiotic cocktail was tested by spreading lysates of 10 midguts on the following growth plates: blood agar plates (tryptic soy agar [TSA] with 5% sheep blood; Remel), MacConkey agar plates (Remel), or LB agar plates (Luria-Bertani agar without antibiotics; KD Medical). Colony counts were assessed 24 h after incubation at 30°C. The same antibiotic concentrations were added to *L. infantum* promastigote cultures maintained in Schneider insect’s medium (Lonza) supplemented with 20% heat-inactivated fetal bovine serum (Gibco) at 27°C. Growth was quantified microscopically over 5 days in antibiotic-treated cultures and controls to test for toxicity against the parasite. Statistical analyses using Student’s *t* test or two-way ANOVA were implemented with PRISM software (La Jolla, CA).

### Data availability.

A full list of taxonomic categories for each species identified in the microbiomes of all conditions, with the means ± standard deviations (SD) of occurrence, is provided in Table S1.
