# Supplementary figures and images for: The Gut Microbiome of the Vector Lutzomyia longipalpis Is Essential for Survival of Leishmania infantum
Source: mBio. 2017 Jan 17;8(1):e01121-16. doi: 10.1128/mBio.01121-16 (PMC5241394; doi:10.1128/mBio.01121-16)

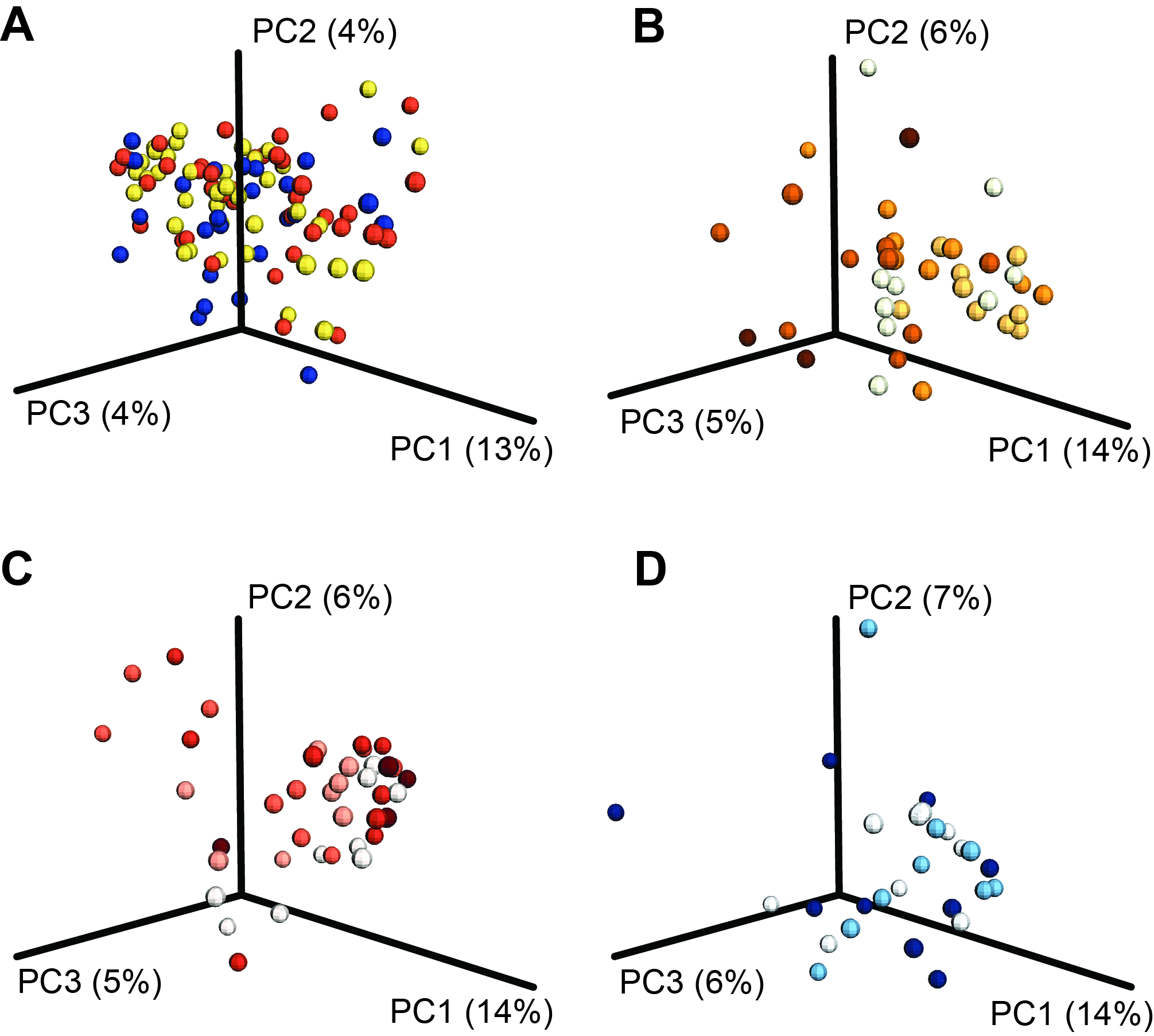

Supplement: Figure S1 [file mbo001173139sf1.tif]

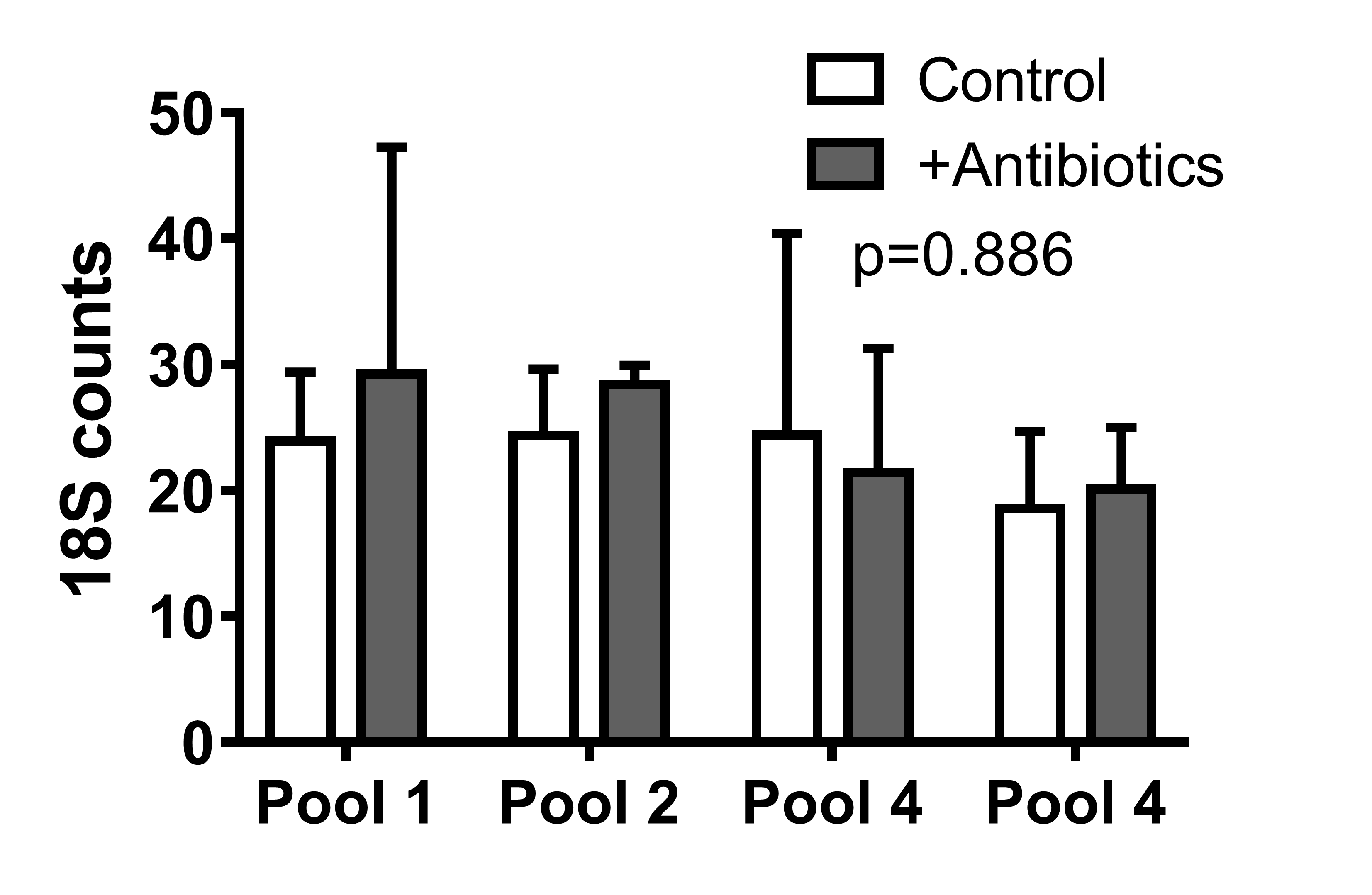

Supplement: Figure S2 [file mbo001173139sf2.tif]

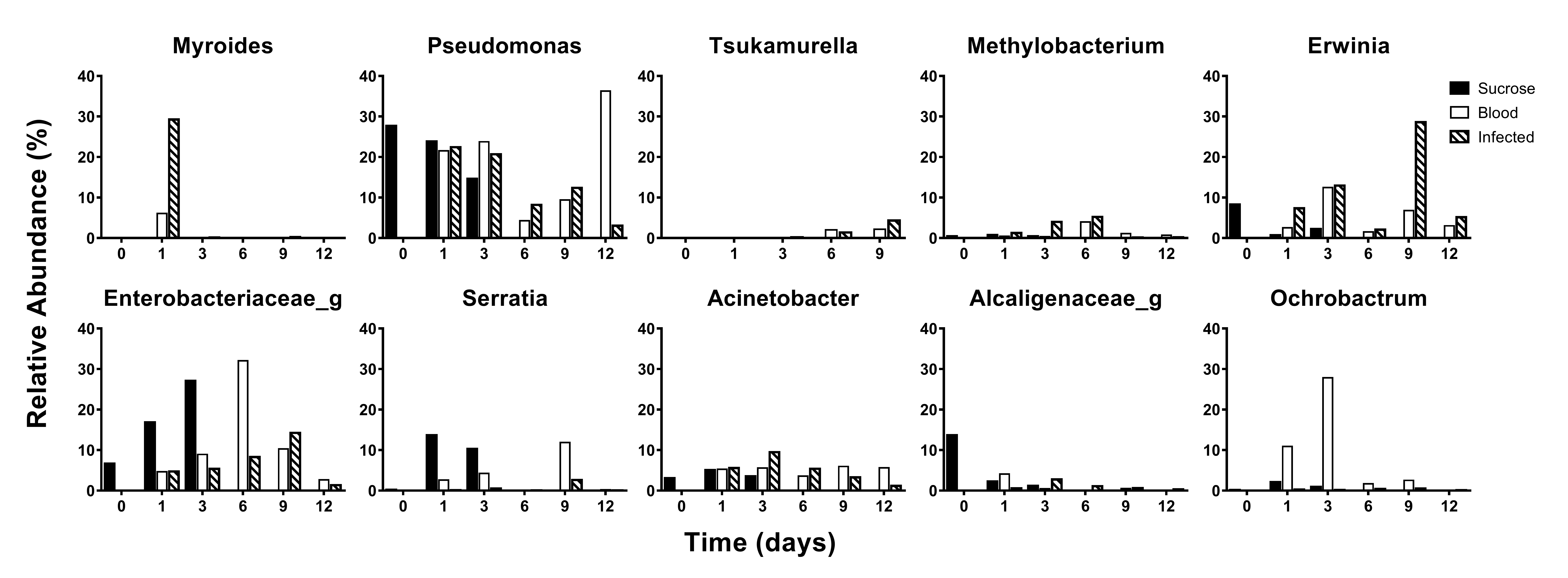

Supplement: Figure S3 [file mbo001173139sf3.tif]
